# CD13 inhibition augments DR4-induced tumor cell death in a p-ERK1/2-independent manner

**DOI:** 10.20892/j.issn.2095-3941.2020.0196

**Published:** 2021-06-15

**Authors:** Jun Ni, Xiaofei Wang, Yue Shang, Yi Li, Shuzhen Chen

**Affiliations:** 1 Department of Cancer Research, Institute of Medicinal Biotechnology, Chinese Academy of Medical Sciences & Peking Union Medical College, Beijing 100050, China

**Keywords:** CD13 inhibition, DR4, TRAIL, neoplasm, therapeutic targets

## Abstract

**Objective:**

Death receptor 4 (DR4; TRAIL-R1) critically mediates extrinsic apoptosis cascades *via* binding to TNF-related apoptosis-inducing ligand (TRAIL). However, intrinsic and/or acquired resistance are observed in the clinical application of TRAIL. The aim of this study was to investigate the function and molecular mechanism of CD13 in the TRAIL/DR4 pathway against tumor cells, and provide a new strategy for improving therapeutic efficacy or overcoming TRAIL-resistance.

**Methods:**

TRAIL protein was expressed as a secretory protein in a *Pichia pastoris* expression system and was isolated and purified by affinity chromatography. The cell viability and apoptosis were evaluated with MTT (thiazolyl blue tetrazolium bromide) assays and annexin V-FITC/PI staining with flow cytometry analysis, respectively. Western blot analysis was used to detect the levels of the indicated proteins in tumor cells. DR4 degradation or stability was examined with cycloheximide chase assays, and cell surface DR4 was assessed with flow cytometric analysis after staining with a FITC-conjugated antibody. The effects of cell migration were determined with Transwell and gelatin zymography assays. A xenograft nude mouse model was used to detect the anti-tumor effect *in vivo*, and the proliferation in tumor tissues was examined with immunohistochemical staining.

**Results:**

CD13 inhibition potently sensitized tumor cells to TRAIL-induced killing, including proliferation inhibition, increased apoptosis, and migration suppression. In addition, the inhibition of CD13 elevated both total cellular expression and cell surface DR4 through stabilizing DR4 by suppressing its degradation. DR4 siRNA attenuated the enhanced anti-tumor effects of TRAIL plus CD13 inhibition. Interestingly, these phenomena were p-ERK1/2 independent, although p-ERK1/2 down-regulation was tightly correlated with the cooperation of TRAIL and CD13 inhibition. Moreover, a synergistic decrease in tumor growth was surprisingly achieved in the xenograft model by treatment of TRAIL with a CD13 inhibitor (**P < 0.01, CDI = 0.47).

**Conclusions:**

CD13 inhibition cooperates with TRAIL in enhancing DR4-mediated cell death, through the up-regulation and stabilization of DR4 in a p-ERK1/2-independent manner. Thus CD13 inhibition has emerged as an effective strategy for TRAIL/DR4-based therapy.

## Introduction

CD13 (also known as aminopeptidase N, APN) is a hydrolytic enzyme that degrades extra-cellular matrix and basement membranes by removing N-terminal neutral amino acids from proteins^1^. This enzyme is closely associated with angiogenesis, invasion, and metastasis in human malignant tumors^2–4^. Emerging evidence indicates that the inhibition of CD13 enzymatic activity—particularly with bestatin, an approved anti-tumor adjuvant drug with good safety, or with monoclonal antibodies including WM15, SJ1D1, and MY7—has antineoplastic effects *via* not only modulation of the immune response but also direct suppressive activity against tumor cells. For instance, CD13 inhibition decreases cell proliferation and induces apoptosis, and these responses are correlated with phosphorylation of MAPK and GSK-3β, decreased cyclin D1 protein expression and degradation of the cellular inhibitor of apoptosis protein-1 (cIAP1) by auto-ubiquitination^5–7^. CD13 inhibitors also participate in decreasing matrix metalloprotein (MMP) enzymatic activity and expression, thereby restraining tumor migration and invasion^8^. In addition, high expression of CD13 in numerous solid tumors and hematologic malignancies has been observed^9^, thus underscoring its advantages as a promising molecular candidate in multi-target clinical oncotherapy.

Death receptor 4 (DR4; also named TRAIL-R1), a member of the DR subgroup of the tumor necrosis factor (TNF) receptor superfamily, is overexpressed in multiple types of tumor cells^10^. DR4 plays an important role in initiating extrinsic cytotoxic signaling cascades through binding of its death domain to ligands such as TNF-related apoptosis-inducing ligand (TRAIL), thus resulting in the assembly of a death-inducing signaling complex^11^. This key mechanism is responsible for the elimination of tumor immunity^12^. Because TRAIL specifically stimulates cell death targeting tumor cells while sparing healthy cells, extensive studies have focused on exploiting agonistic antibodies against DR and recombinant TRAIL protein to achieve high therapeutic potential. To date, dulanermin (AMG 951), a recombinant soluble TRAIL, is in an ongoing phase III trial, and phase I and phase II trials have been completed^13,14^. In addition, 7 tests have been performed on DR agonistic monoclonal antibodies, including mapatumumab (HGS-ETR1), the only DR4 targeting antibody assessed in phase I/II trials^15^. Although these treatments have exhibited efficacy in triggering apoptosis for sensitizing tumor cells to radiotherapy, targeted therapy and chemotherapy, the outcomes have been disappointing, owing to the development of intrinsic and/or acquired resistance in many tumor cell lines; this drawback remains a crucial challenge for researchers^16,17^. Hence, determining how to restore the sensitivity of tumor cells to TRAIL/DR activity is urgently necessary to improve therapeutic efficacy or overcome TRAIL-resistance.

Given the potential of DR and CD13 inhibition, the current study demonstrated a novel molecular mechanism through which inhibition of CD13 facilitates tumor cell sensitization to TRAIL/DR4-induced cell death, thus extending understanding of the extrinsic cytotoxic pathway in tumor cells and providing a new strategy for TRAIL-based treatment.

## Materials and methods

### Cell lines and cell culture

The lung adenocarcinoma cell line A549, breast adenocarcinoma cell line MCF-7, fibrosarcoma cell line HT1080, and pancreatic carcinoma cell line PANC-1 were stored in our laboratory. A549 and HT1080 sells were cultured in RPMI 1640 medium (HyClone, Thermo Fisher Scientific, Waltham, MA, USA), and MCF-7 and PANC-1 cells were cultured in DMEM (HyClone, Thermo Fisher Scientific, Waltham, MA, USA) supplemented with 10% FBS (HyClone, Thermo Fisher Scientific, Waltham, MA, USA), 100 U/mL penicillin, and 0.1 mg/mL streptomycin (Solarbio Science & Technology, Beijing, China) at 37 °C in a humidified atmosphere containing 5% CO_2_.

### Reagents

12-O-tetradecanoylphorbol-13-acetate (TPA) was purchased from Cell Signaling Technology (Danvers, MA, USA). MG132 and PD98059 were purchased from MedChemExpress (Monmouth Junction, NJ, USA). zVAD-fmk was purchased from Selleck (Houston, TX, USA).

### CD13 inhibition treatment

Cells were treated with bestatin (National Institutes for Food and Drug Control, Beijing, China), WM15 (Abcam, Cambridge, MA, USA), or CD13 siRNAs (#1: target 5′-CAAGAACGCCAACAGCTCC-3′, #2: target 5′-CACCTTGGACCAAAGTAAA-3′) at the indicated concentrations, and this was followed by cell proliferation assays and Western blot analysis.

### TRAIL protein expression and purification

TRAIL protein was constructed in our laboratory and expressed as a secretory protein in a *Pichia pastoris* expression system. TRAIL expressing yeast strains (40 μL) were inoculated into 100 mL BMGY medium (1% yeast extract, 1% glycerol, 100 mmol/L potassium phosphate buffer, pH 6.0, 2% tryptone, 0.00004% biotin, and 1.34% YNB) in a shaking incubator (220 rpm) at 30 °C for 36 h. Yeast cells were precipitated after standing for 4–6 h and re-suspended in 100 mL BMMY medium (1% yeast extract, 1.5% methanol, 100 mmol/L potassium phosphate buffer, pH 6.0, 6% tryptone, 0.00004% biotin, and 1.34% YNB) with added methanol to 1.5% every 24 h in a shaking incubator (220 rpm) at 26 °C for 72 h. Supernatant was obtained after centrifugation (7,800 × *g*, 4 °C, 15 min), and TRAIL protein was isolated and purified with Ni^2+^ affinity chromatography (His-Trap HP, GE Healthcare, Pittsburgh, Pennsylvania, USA). Protein concentrations were detected with BCA assays (Thermo Fisher Scientific, Waltham, MA, USA).

### Cell proliferation assays

Cells seeded in 96-well plates at 2 × 10^3^ cells per well in 100 μL culture medium were treated with the tested agents on the second day after seeding. MTT solution (20 μL of 5 mg/mL solution) was added to the cell supernatants at the indicated time points and incubated at 37 °C for an additional 4 h. After removal of the medium, 200 μL of DMSO was added to each well. The absorbance at 570 nm was measured with a microplate reader (Thermo Fisher Scientific, Waltham, MA, USA). The cell viability was normalized to that of the untreated cells.

### Western blot analysis

Harvested cells were lysed with RIPA lysis buffer (150 mmol/L NaCl, 50 mmol/L Tris-HCl pH 8.0, 2% NP-40, 0.5% sodium deoxycholate, and 0.2% SDS) containing protease inhibitor (Beyotime, Jiangsu, China). A BCA assay kit (Thermo Fisher Scientific, Waltham, MA, USA) was used to determine the protein concentrations in the cell lysates. Briefly, the proteins were separated by 10% SDS-PAGE electrophoresis, then electrotransferred onto PVDF membranes. After being blocked with 5% bovine serum albumin for 1 h, the membranes were incubated with primary antibodies at 4 °C overnight and secondary antibodies for 1 h at room temperature. The following primary antibodies were used: anti-p44/42 MAPK (ERK 1/2), anti-phospho-p44/42 MAPK (ERK 1/2), anti-PARP (Cell Signaling Technology, Danvers, MA, USA), anti-human CD261 azide free (DR4) (Diaclone, Besancon, France), anti-DR5 (ProSci, San Diego, California, USA), and anti-CD13 (Santa Cruz Biotechnology, Dallas, Texas, USA). The secondary antibodies were anti-GAPDH (1C4) and anti-β-tubulin monoclonal antibodies (AmeriBiopharma, Wilmington, Delaware, USA). Enhanced chemiluminescence (Merck Millipore, Burlington, MA, USA) was used to detect protein expression. Quantification of target proteins was calculated by intensity scanning, and the results were normalized to the intensity of the relevant internal control protein. Data are reported as mean ± SD for 3 independent experiments.

### Detection of apoptosis

Apoptosis was evaluated with an annexin V-FITC/PI apoptosis detection kit (BioFriend Biotechnology, Beijing, China) according to the manufacturer’s instructions. Detection of PARP cleavage with Western blot analysis was used as an additional indicator of apoptosis. Briefly, after 24 h of drug treatment, cells were washed with phosphate-buffered saline (PBS), and the harvested cells were incubated with 500 μL of binding buffer containing 5 μL of annexin V-FITC and 10 μL of PI at room temperature for 15 min. Flow cytometry was used to measure the apoptosis rates.

### Detection of cell surface DR4 (TRAIL-R1)

After 24 h of treatment with different concentrations of the indicated agents, cells were harvested by trypsinization and washed twice with PBS. Next, the cells were incubated for 20 min at 4 °C with mouse anti-human CD261 azide free (DR4) primary antibody (Diaclone, Besancon, France) or normal mouse IgG control (Santa Cruz Biotechnology, Dallas, Texas, USA) diluted 1:25 in PBS. After being washed twice with PBS containing 1% FBS, cells in 50 μL PBS containing 1% FBS were incubated with 2 μL FITC-goat anti-mouse IgG secondary antibody (Bioss, Beijing, China) for 30 min at room temperature. Then the cells were washed twice and resuspended in 300 μL PBS containing 1% FBS. Flow cytometry was used for detection. Analysis was performed with FACScan software.

### Silencing of DR4 (TRAIL-R1) with small interfering RNA (siRNA)

DR4 siRNAs (#1: target 5′-AACGAGATTCTGAGCAACG CA-3′, #2: target 5′-AATGAGATCGATGTGGTCAGA-3′) and negative control siRNA were purchased from Ruibo Biotechnology (Guangzhou, China). Cells were transfected with these siRNAs according to the instructions of the Ruibo FECT™ CP transfection kit. Twenty-four hours after transfection, cells were detected with Western blot analysis or used for cell proliferation assays.

### Transwell assays

Serum-free medium (100 μL) containing cells (2–5 × 10^4^ cells/well) was added to the upper chambers of 24-well Transwell filter inserts (8-μm pore size) (Corning, NY, USA). Medium (600 μL) containing 20% FBS was added to the bottom chambers. After treatment for 24 h at 37 °C, the cells in the upper chambers were removed, and the cells at the bottom of the membranes were fixed with methanol for 15–30 min. Then the cells were stained with crystal violet for 10 min (Solarbio Science & Technology, Beijing, China). After images were captured by microscopy, the insert membranes along the edge of the chambers were cut and placed in 96-well plates with 33% acetic acid (150 μL) and subjected to low-speed vibration for 10 min. The membranes were carefully discarded after crystal violet was fully dissolved in the plates. The absorbance values at 570 nm were measured with a microplate reader (Thermo Fisher Scientific, Waltham, MA, USA), and the results were normalized to those of the control group.

### Gelatin zymography

After drug treatment for 12 h, cells (1 × 10^6^ cells/well) in 6-well plates were incubated in serum-free medium for an additional 24 h. The conditioned medium was collected and separated with 10% SDS-PAGE containing 1 mg/mL gelatin. After electrophoresis, the gels were washed with dH_2_O and soaked twice in 2.5% Triton X-100 solution for 40 min at room temperature. Then, the gels were incubated in substrate buffer solution (10 mmol/L CaCl_2_, 50 mmol/L Tris, 200 mmol/L NaCl, and 1 μmol/L ZnCl_2_, pH 7.5) at 37 °C for 42 h; negatively stained with 0.1% Coomassie blue in 45% methanol and 10% acetic acid for 4 h; and washed in destaining solution (methanol: dH_2_O:acetic acid = 45:45:10) for 1–2 h to visualize the bands corresponding to MMP activity. The bands were detected with an image acquisition and analysis system (ProteinSimple, CA, USA).

### Detection of CD13/aminopeptidase activity

Cells were seeded in 96-well plates at 2 × 10^5^/mL in 180 μL culture medium per well and treated with the tested agents (20 μL) for 1 h at 37 °C. Then 20 μL of the protease substrate L-alanine 4-nitroanilide hydrochloride (20 mmol/L) (Sigma-Aldrich, St. Louis, MO, USA) was added and incubated at 37 °C for 1 h. Absorbance values at a wavelength of 450 nm were measured with a microplate reader (Thermo Fisher Scientific, Waltham, MA, USA). CD13 activity was calculated relative to that in untreated cells.

### RNA extraction and quantitative real time PCR analysis

Total RNA was extracted with a Total RNA Purification Kit (Shanghai Feijie, Shanghai, China) according to the manufacturer’s instructions. RNA quality and quantity were assessed with a NanoDrop^®^ Lite UV-vis spectrophotometer (Kaiao, Beijing, China). cDNA was synthesized with ReverTra Ace qPCR RT Master Mix (Toyobo Co., Ltd., Osaka, Japan). The mRNA levels of DR4, DR5, and GAPDH were measured *via* quantitative real time PCR with SYBR Premix ExTaq (Takara, Tokyo, Japan) and an ABI 7500HT Fast Real-time PCR system (Applied Biosystems, Foster City, CA, USA). The following primers were used: DR4 5′-GCGGGGAGGATTGAACC AC-3′ (forward) and 5′-CGACGACAAACTTGAAGGTCTT-3′ (reverse); and GAPDH 5′-TTCTATAAATTGAGCCCGCA GCC-3′ (forward) and 5′-CCGTTGACTCCGACCTTCAC-3′ (reverse). GAPDH served as a normalization control. The relative fold-change values were analyzed with the 2^-ΔΔCt^ method.

### *In vivo* models

Fibrosarcoma HT1080 cells (0.8 × 10^7^ cells in 200 μL PBS) were inoculated subcutaneously into the right armpits of 6-week-old female BALB/c athymic nude mice (SPF Biotechnology, Beijing, China). Twenty-one days later, the growing solid tumors were explanted into the right armpits of other mice, which were randomly divided into 4 groups (6 per group). Mice were treated with TRAIL (10 mg/kg, on days 1, 8, and 15, *via* tail vein injection) or bestatin (20 mg/kg, for 5 consecutive days per week, *via* oral administration). Body weights and tumor volumes were monitored every 2 days during the treatment process. After the mice were sacrificed at the end of the experiment, neoplastic blocks were dissected and weighed, then prepared for Western blot analysis, or formalin fixation and paraffin embedding for immunohistochemistry analysis. The tumor volume was calculated according to the formula: V = ab^2^/2 (where a and b represent the length and the width of the tumor, respectively). The coefficient of drug interaction (CDI) was used to evaluate the mode of drug interaction, according to the formula CDI = AB/A × B (where AB is the survival rate of the combined treatment group relative to the control group, and A or B is the survival rate for the single-drug group relative to the control group). CDI < 1.0 indicated a synergistic effect, CDI < 0.7 indicated a strongly synergistic effect^17^.

### Immunohistochemistry

After dewaxing and antigen retrieval, tissue sections were quenched with endogenous peroxidase blockers for 10 min at room temperature. After being blocked with 5% bovine serum albumin (in PBS) for 30 min at 37 °C, sections were incubated with anti-Ki67 (ZSGB-BIO, Beijing, China) at 4 °C overnight. A universal DAB detection kit (ZSGB-BIO, Beijing, China) was used to stain Ki67 antibody bound tissues. Immunostaining was observed in 3 randomly selected fields under a microscope (Leica QWin Standard). The images were photographed and analyzed with Leica QWin analytical software. The percentage of cells staining positive for Ki67 expression were normalized to the values in the control group.

### Statistical analysis

The results are presented as mean ± SD. Statistical analysis and graphing were performed in GraphPad Prism 8 (GraphPad Software, Inc., La Jolla, CA, USA). Student’s *t*-test/one-way ANOVA and related nonparametric tests were conducted to evaluate the statistical significance, and the threshold for significance was set at *P* < 0.05.

### Ethical approval

All experiments involving animals were performed in accordance with the relevant guidelines and regulations (Approval No. IMB-20180510D7).

## Results

### CD13 inhibition and TRAIL have cooperative tumor-suppressive effects toward human tumor cells

**Figure 1:**
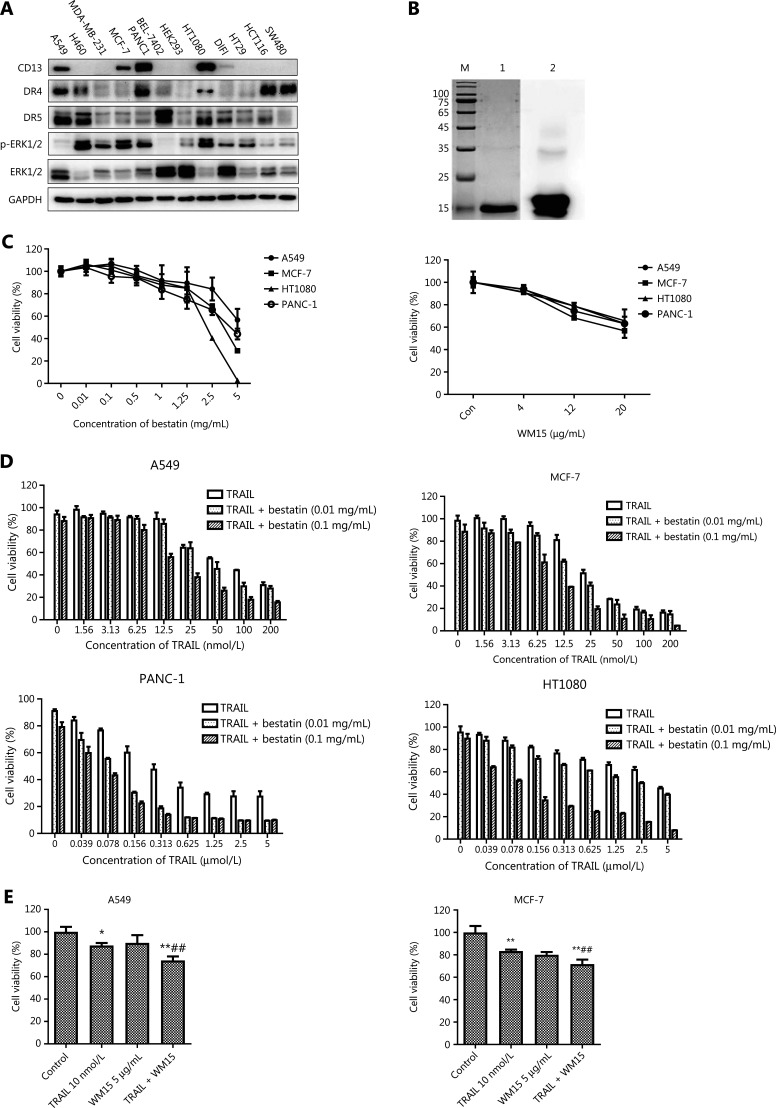
CD13 inhibition enhances the effects of TRAIL in decreasing cell survival in tumor cells. (A) Expression levels of CD13, DR4, DR5, ERK1/2, and p-ERK1/2 in the indicated tumor cell lines, determined by Western blot analysis. (B) Analysis of the purification of TRAIL protein. M: Molecular weight protein markers (kDa); 1: TRAIL protein; 2: anti-TRAIL Western blot. (C) The tumor cell lines (A549, MCF-7, HT1080, and PANC-1) in 96-well plates were exposed to bestatin (0.01, 0.1, 0.5, 1, 1.25, 2.5, or 5 mg/mL) or WM15 (4, 12, or 20 μg/mL) for 48 h. (D) The indicated cell lines in 96-well plates were co-treated with TRAIL and bestatin for 48 h: A549, TRAIL 1.56–200 nmol/L, bestatin 0.01 or 0.1 mg/mL; MCF-7, TRAIL 1.56–200 nmol/L, bestatin 0.01 or 0.1 mg/mL; HT1080, TRAIL 0.039–5 μmol/L, bestatin 0.01 or 0.1 mg/mL; and PANC-1, TRAIL 0.039–5 μmol/L, bestatin 0.01 or 0.1 mg/mL. (E) The indicated cell lines were pretreated with WM15 (5 μg/mL) for 8 h and then co-treated with TRAIL (10 nmol/L) for an additional 48 h. The survival rates were normalized to the control group values (untreated cells). Data are means ± SD for 3 independent experiments. *P < 0.05 and **P < 0.01 *vs*. control, ^##^*P* < 0.01 *vs*. TRAIL.

Because MEK/ERK signaling regulates DR4 expression in different tumor cell lines^18^, to study the correlation between CD13-inhibition and death receptors, we first detected the levels of CD13, DR4, DR5, ERK1/2, and phosphorylated ERK1/2 in 13 indicated tumor cell lines by Western blot analysis (**Figure 1A**), and calculated the relative protein levels (**Supplementary Figure S1**). The expression levels of all the detected proteins varied in the different cells, and no clear correlation was observed between the expression of CD13 with DRs as well as ERK1/2 and its phosphorylated form in these tumor cell lines. According to the expression of CD13, 4 cell lines highly CD13-expressing (A549, MCF-7, HT1080, and PANC-1) were selected for treatment with CD13 inhibitors in follow-up experiments. Bestatin (a CD13 enzyme inhibitor) and WM15 (an anti-CD13 antibody) were used as inhibitors of CD13. After a 2-day exposure, bestatin (0.01–5 mg/mL) and WM15 (4, 12, and 20 μg/mL) both inhibited cell proliferation in the 4 tumor cell lines in a dose-dependent manner (**Figure 1C**), thus indicating that CD13 inhibition restrains the proliferation of human tumor cells.

Next, TRAIL protein was expressed as a secretory protein in a *Pichia pastoris* expression system, and was isolated and purified with affinity chromatography. Expression and verification of TRAIL (with an anti-TRAIL antibody) indicated successful expression of TRAIL protein with high purity (**Figure 1B**). As shown in **Figure 1D**, TRAIL inhibited the proliferation of the tested cell lines in a dose-dependent manner, and bestatin at 0.01 and 0.1 mg/mL slightly inhibited the growth of the cell lines, whereas bestatin at the indicated concentrations prominently enhanced the inhibitory effects of TRAIL. A549 (TRAIL: IC_50_ = 70.87 nmol/L, TRAIL + bestatin-0.1 mg/mL: IC_50_ = 18.43 nmol/L) and MCF-7 (TRAIL: IC_50_ = 29.91 nmol/L, TRAIL + bestatin-0.1 mg/mL: IC_50_ = 8.77 nmol/L) were more sensitive to bestatin plus TRAIL treatment. Moreover, similarly to our observations with bestatin, WM15 augmented the effects of TRAIL in decreasing cell viability rates (^##^*P* < 0.01) (**Figure 1E**), thus suggesting that CD13 inhibition effectively facilitates tumor cell sensitization to TRAIL-induced cell death. Given the hydrolytic enzyme activity of CD13, we also detected whether the cooperation of CD13 inhibition and TRAIL might exhibit this synergistic effect *via* altering CD13 activity; unexpectedly, compared with that in the bestatin-treated group, the CD13 activity did not significantly differ in the presence of TRAIL and bestatin (**Supplementary Figure S2**).

Bestatin has been reported to repress the invasive potential of the human osteosarcoma cell lines MG63 and HOS^19^. Here, we explored the anti-invasion effects of TRAIL/DR4 and CD13 inhibition in highly invasive A549 and HT1080 cells. As compared with the results in the control or single treatment groups, TRAIL plus bestatin markedly decreased the invasiveness of the 2 tested cell lines (**P < 0.01; ^##^*P* < 0.01) without affecting cell viability (**Supplementary Figure S3A**). To investigate the underlying mechanisms, we next assessed the enzymatic activity and expression of MMP and tissue inhibitor of metalloproteinases (TIMP) by gelatin zymography and Western blot analysis. The MMP-2 activity decreased and the TIMP-1 levels increased in both A549 and HT1080 cells 24 h after exposure to TRAIL and bestatin (**Supplementary Figure S3B** and **S3C**), thus suggesting that decreased MMP activity might be involved in this anti-invasion effect. Together, these data significantly indicate that CD13 inhibition cooperates with TRAIL in exerting tumor-suppressive effects through suppressing proliferation and invasion in human tumor cells.

### CD13 inhibition sensitizes human tumor cells to TRAIL-induced apoptosis

**Figure 2:**
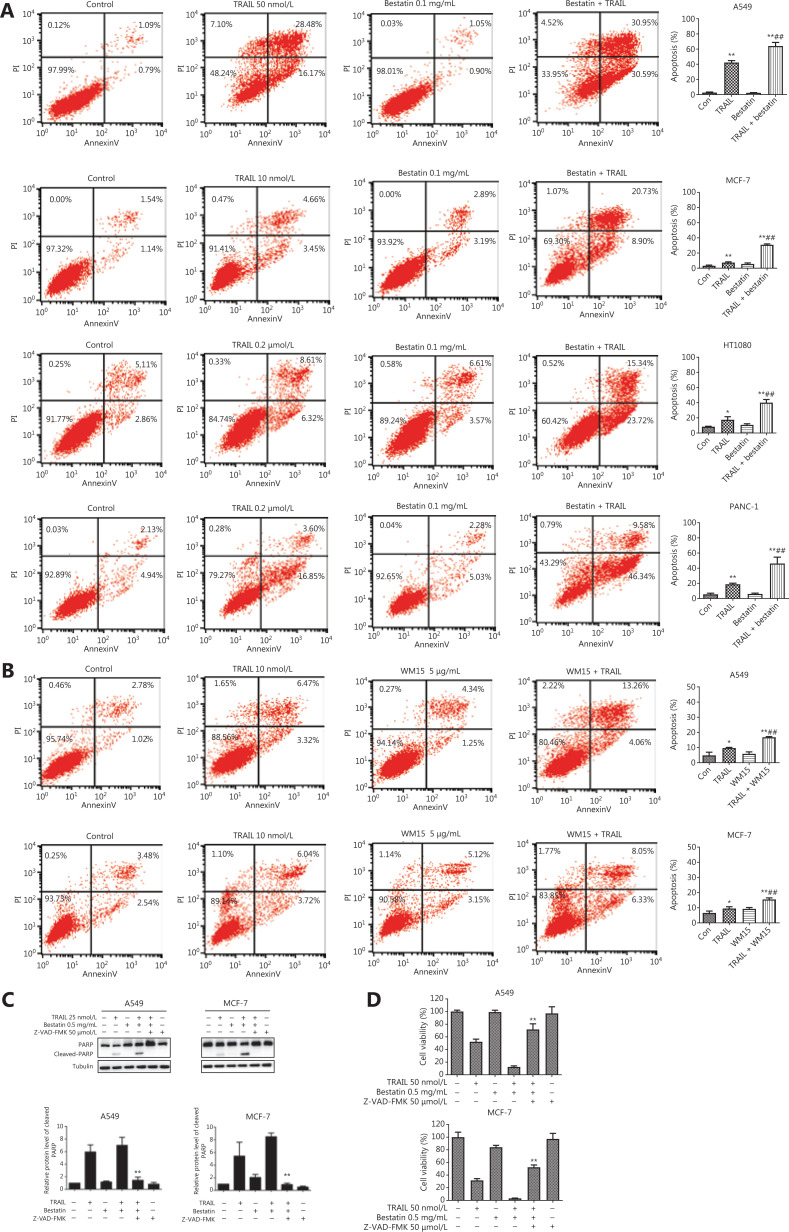
CD13 inhibition sensitizes human tumor cells to TRAIL-induced apoptosis. (A) The indicated cell lines were co-treated with TRAIL and bestatin for 24 h: A549, TRAIL 50 nmol/L, bestatin 0.1 mg/mL; MCF-7, TRAIL 10 nmol/L, bestatin 0.1 mg/mL; HT1080, TRAIL 0.2 μmol/L, bestatin 0.1 mg/mL; and PANC-1, TRAIL 0.2 μmol/L, bestatin 0.1 mg/mL. (B) The indicated cell lines were exposed to WM15 (5 μg/mL) for 8 h, then co-treated with TRAIL (10 nmol/L) for an additional 24 h. Apoptosis was detected with annexin V-FITC/PI staining and flow cytometry analysis. The apoptotic rate was the sum of the percentages of early and late apoptotic cells. *P < 0.05 and **P < 0.01 *vs.* control, ^##^*P* < 0.01 *vs.* TRAIL. (C) The cell lines were exposed to TRAIL (25 nmol/L), bestatin (0.5 mg/mL) and zVAD.fmk (50 μmol/L) (zVAD.fmk pretreatment for 30 min) for 12 h. Quantification of the indicated proteins was calculated, and each was previously normalized to the level of tubulin. **P < 0.01 *vs.* TRAIL + bestatin. (D) The indicated cell lines were treated with TRAIL (50 nmol/L), bestatin (0.5 mg/mL), and zVAD.fmk (50 μmol/L) (zVAD.fmk pretreatment for 30 min) for 48 h. **P < 0.01 *vs.* TRAIL + bestatin. Data are means ± SD for 3 independent experiments.

To further determine whether CD13 inhibitors might potentiate the anti-tumor activity of TRAIL *via* TRAIL-induced apoptosis, we assessed the apoptosis of cells treated with TRAIL in the absence or presence of bestatin or WM15. Annexin V-FITC/PI staining and flow cytometry analysis showed that bestatin/WM15 significantly increased the rates of apoptosis caused by TRAIL in all indicated tumor cell lines (^##^*P* < 0.01) (**Figure 2A and 2B**). For example, in A549 cells, TRAIL alone at 50 nmol/L induced 35%–45% apoptosis, and bestatin alone at 0.1 mg/mL induced only 2% apoptosis; however, TRAIL plus bestatin caused more than 70% apoptosis (**Figure 2A**). Furthermore, bestatin enhanced PARP cleavage, a hallmark of cell apoptosis, in both A549 and MCF-7 cells exposed to TRAIL simultaneously; and then the increased expression of cleaved-PARP induced by TRAIL plus bestatin was abolished by zVAD.fmk, an irreversible pan-caspase inhibitor (**P < 0.01) (**Figure 2C**). In addition, as anticipated, zVAD.fmk attenuated the synergistic decrease in cell viability caused by bestatin and TRAIL (**P < 0.01) (**Figure 2D**). These data together clearly suggest that CD13 inhibition potently sensitizes tumor cells to TRAIL-mediated killing through augmenting TRAIL-induced apoptosis.

### CD13 inhibition increases the expression and cell surface of DR4 protein through stabilizing DR4

**Figure 3:**
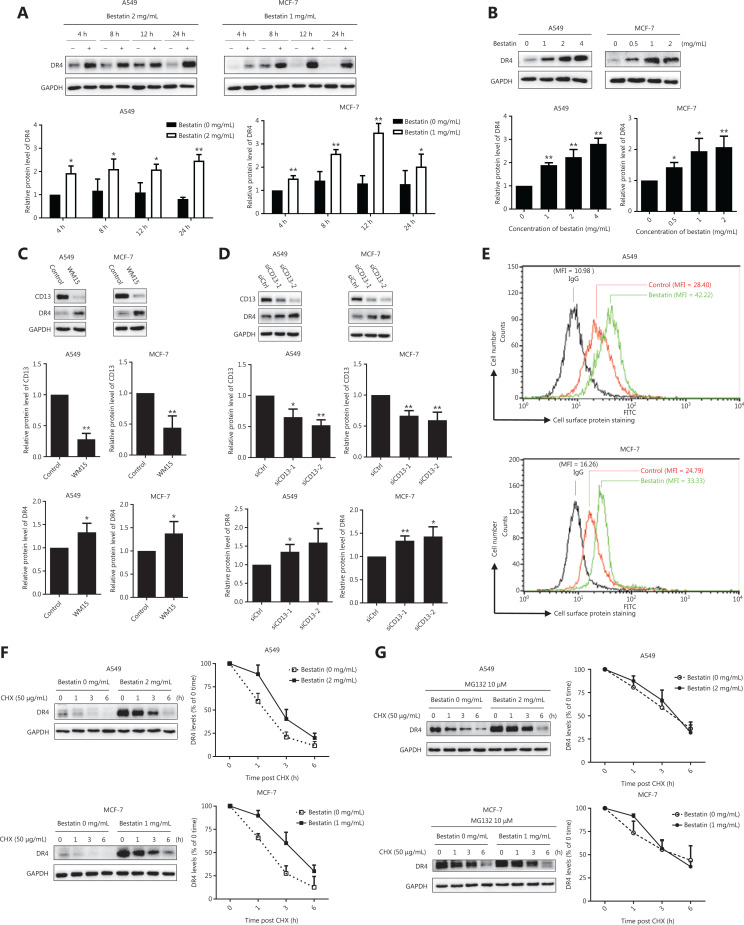
CD13 inhibition increases the expression of DR4 protein and cell surface DR4, and suppresses DR4 degradation. (A) The indicated cell lines were treated with bestatin (A549: 2 mg/mL, MCF-7: 1 mg/mL) for 4, 8, 12, or 24 h; (B) The indicated cell lines were treated with bestatin (A549: 1, 2, or 4 mg/mL, MCF-7: 0.5, 1, or 2 mg/mL) for 24 h. (C) The indicated cell lines were exposed to WM15 (5 μg/mL) for 24 h. (D) The indicated cell lines were exposed to CD13 siRNA (50 nmol/L) for 24 h. (E) The indicated cell lines were treated with bestatin (A549: 4 mg/mL, MCF-7: 2 mg/mL) for 24 h and then harvested for staining of DRs and subsequent flow cytometric analysis of cell surface DR4. The control cells were stained with a matched control FITC-conjugated IgG isotype antibody or FITC-conjugated anti-DR4 antibody, and the bestatin-treated cells were stained with FITC-conjugated anti-DR4 antibody. The MFI for each sample is indicated. (F) The indicated cell lines were exposed to bestatin (A549: 2 mg/mL, MCF-7: 1 mg/mL) for 24 h, and this was followed by the addition of 50 μg/mL CHX at different times. (G) Cells were pretreated with MG132 (10 μM) for 2 h and then received the indicated treatments for different times. Whole cell lysates were prepared from these cells and used to detect the levels of the indicated proteins with Western blot analysis. The results are plotted as the DR4 levels relative to those at time 0 of CHX treatment. The indicated proteins were quantified, and each was previously normalized to the level of GAPDH. Data are means ± SD for 3 independent experiments. *P < 0.05 and **P < 0.01 *vs.* control.

To understand the mechanisms underlying the synergistic anti-tumor effects of TRAIL and CD13 inhibition, we further determined whether CD13 inhibitors might regulate the expression of the TRAIL receptors DR4 and DR5, which are essential components in the TRAIL-induced apoptosis pathway^20^. As shown in **Figure 3A and 3B**, bestatin clearly increased the DR4 protein levels in a time- and dose-dependent manner, and this response was accompanied by a decrease in CD13 (*P < 0.05; **P < 0.01). Moreover, DR4 expression was up-regulated by WM15 in the tested cell lines (*P < 0.05) (**Figure 3C**). Furthermore, CD13 siRNAs were used to knock down CD13 expression to examine its effects on DR4 expression. As expected, siCD13 not only blocked CD13 expression but also markedly elevated DR4 protein levels (*P < 0.05; **P < 0.01) (**Figure 3D**). However, bestatin did not alter the mRNA levels of DR4 in both A549 and MCF-7 cells (**Supplementary Figure S4**).

Moreover, because DR4 has biological activity at the cell surface^21^, we detected cell surface DR4 in tumor cells through flow cytometric analysis after staining with a FITC-conjugated antibody. After a 24 h exposure, in A549 cells, the mean fluorescence intensity (MFI) of the control group was 28.40, whereas that of the bestatin treatment group was as high as 42.22 (**Figure 3E**). In addition, in MCF-7 cells, the MFI increased from 24.79 in control cells to 33.33 in bestatin-treated cells (**Figure 3E**). Together, these results suggest that CD13 inhibition prominently increases DR4 on tumor cell surfaces, as evidenced by a rightward shift in the peak positions. In contrast, bestatin decreased cell surface expression of DR5 in the tested cells under the same conditions (**Supplementary Figure S5**).

Next, to determine the mechanism of the DR4 increase mediated by CD13 inhibitors, we observed the effects of bestatin on the modulation of DR4 stability and degradation by conducting post-cycloheximide (CHX) chase assays. As shown in **Figure 3F**, the rate of DR4 degradation was much slower in bestatin-treated cells than in control cells at all timepoints examined (1, 3, and 6 h) in both A549 and MCF-7 cells, thereby indicating that CD13 inhibition stabilizes DR4 or suppresses DR4 degradation. Interestingly, the proteasome inhibitor MG132 accentuated this slow degradation process (**Figure 3G**), thus suggesting that the DR4 degradation regulated by CD13 inhibition is involved in the ubiquitin-proteasome pathway. Together, our results indicated that CD13 inhibition increases DR4 levels, including those at the cell surface, through maintaining protein stability in tumor cells.

### The critical role of DR4 elevation in the enhancement of TRAIL-mediated killing by CD13 inhibition

**Figure 4:**
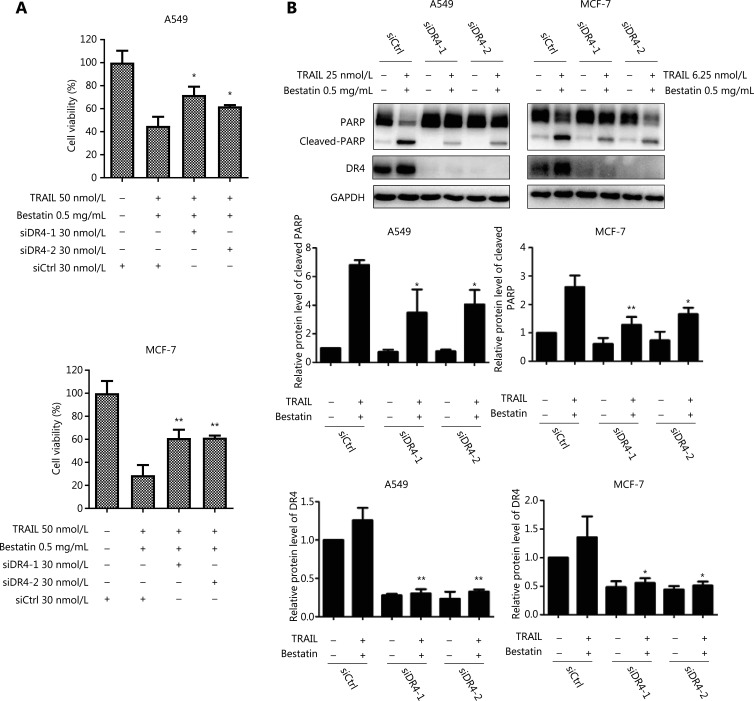
Loss of DR4 protects tumor cells against enhanced killing effects of TRAIL together with CD13 inhibition. (A) A549 and MCF-7 cells were exposed to 48 h pretreatment with 30 nmol/L DR4 siRNA, followed by 24 h treatment with TRAIL (50 nmol/L) and bestatin (0.5 mg/mL). (B) The expression of the indicated proteins was assessed by Western blot analysis after 36 h pretreatment with DR4 siRNA followed by 24 h treatment with TRAIL and bestatin at the indicated concentrations. Data are means ± SD for 3 independent experiments. *P < 0.05 and **P < 0.01 *vs.* TRAIL + bestatin + siCtrl.

Because binding of TRAIL to its receptors, including DR4, is an essential step in inducing apoptotic signaling, we observed the increased DR4 levels after bestatin treatment. However, the role of DR4 elevation in regulating the increase in TRAIL-induced cell death in the presence of CD13 inhibition is unknown. To investigate this role, we blocked induction of DR4 through siRNA silencing and compared the killing effects mediated by bestatin plus TRAIL in the absence and presence of siDR4. Three pairs of siRNA sequences were designed for DR4; among them, the 2 siRNAs with the strongest inhibitory potential were selected for follow-up studies. As presented in **Figure 4A**, bestatin plus TRAIL was still effective in decreasing cell viability, whereas siDR4-1 or siDR4-2 attenuated this effect, thereby significantly increasing cell growth (*P < 0.05; **P < 0.01). In addition, we found increased levels of cleaved PARP in control cells exposed to TRAIL and bestatin, but only a minimal increase in DR4-knockdown A549 and MCF-7 cells subjected to the same treatment (*P < 0.05; **P < 0.01) (**Figure 4B**). Overall, the loss of DR4 sufficiently protected tumor cells from undergoing apoptosis enhancement by TRAIL in cooperation with CD13 inhibition, thus demonstrating a key role of DR4 elevation in augmenting TRAIL-induced cell death in the presence of CD13 inhibition.

### Down-regulation of p-ERK1/2 involves an increase in TRAIL-mediated killing in the presence of CD13 inhibition

**Figure 5:**
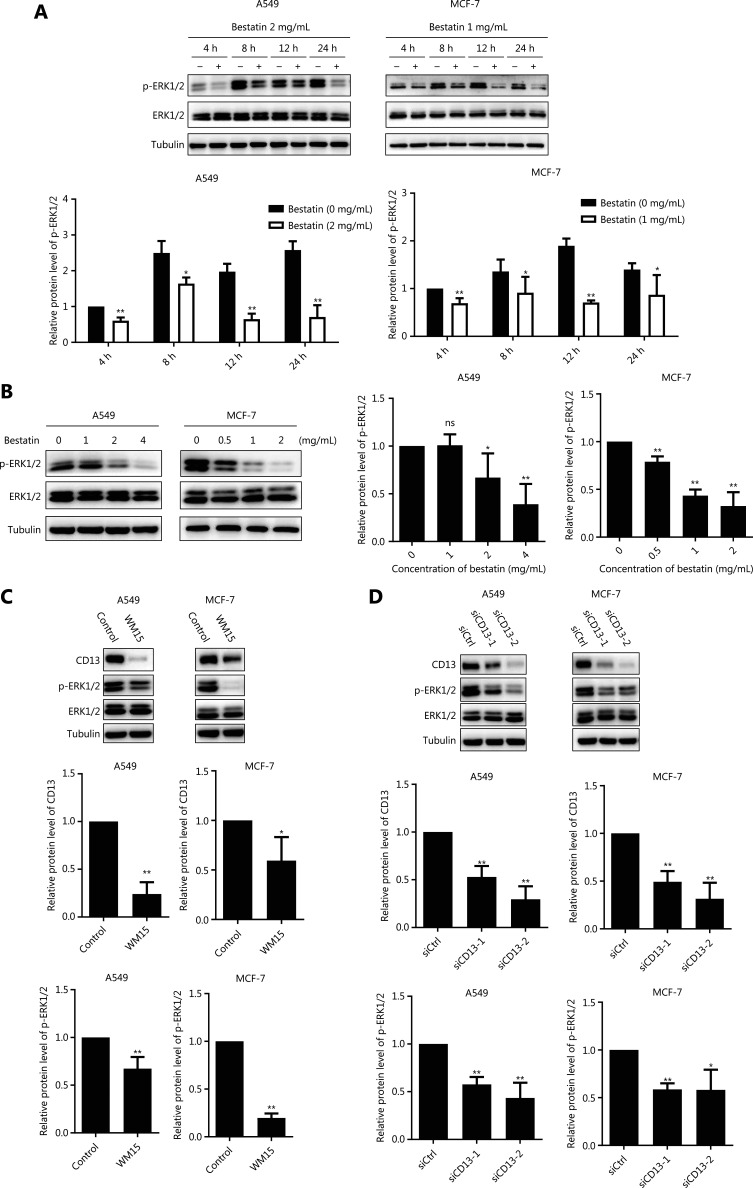
CD13 inhibition decreases ERK1/2 phosphorylation in tumor cells. (A) The indicated cell lines were treated with bestatin (A549: 2 mg/mL, MCF-7: 1 mg/mL) for 4, 8, 12, or 24 h. (B) The indicated cell lines were treated with bestatin (A549: 1, 2, or 4 mg/mL, MCF-7: 0.5, 1, or 2 mg/mL) for 24 h. (C) The indicated cell lines were exposed to WM15 (5 μg/mL) for 24 h. (D) The indicated cell lines were exposed to CD13 siRNA (50 nmol/L) for 24 h. The indicated proteins were quantified, and each was previously normalized to the level of tubulin. Data are means ± SD for 3 independent experiments. *P < 0.05 and **P < 0.01* vs.* control.

**Figure 6:**
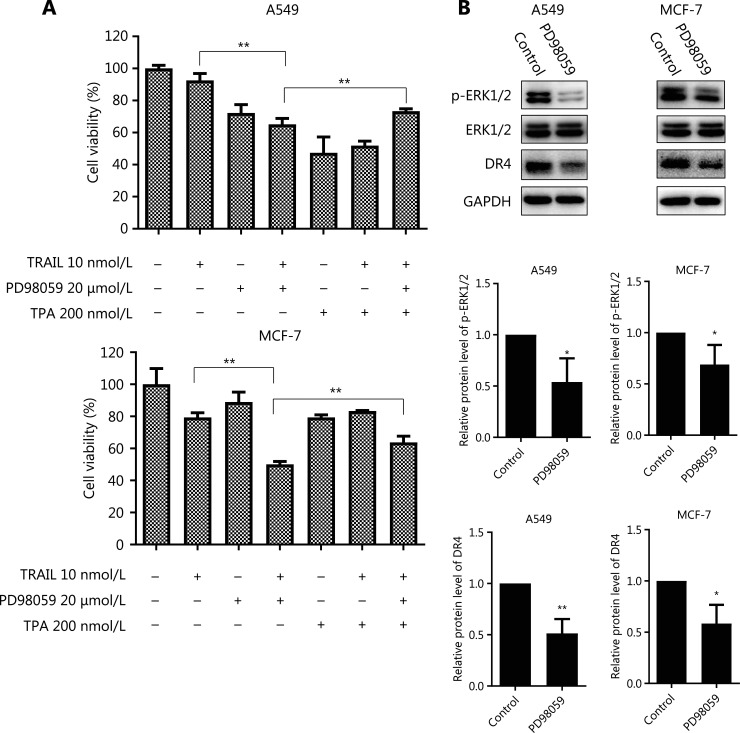
Inhibition of ERK1/2 phosphorylation augments the cell death caused by TRAIL without up-regulating DR4. (A) The indicated cell lines in 96-well plates were pretreated with PD98059 (20 μmol/L) for 6 h followed by treatment with TPA (200 nmol/L) for 30 min and co-treatment with TRAIL (10 nmol/L) for an additional 48 h. The survival rates were normalized to the control group values (untreated cells). **P < 0.01* vs.* TRAIL + PD98059. (B) The indicated cell lines were treated with PD98059 (20 μmol/L) for 24 h, and then the whole cell lysates were prepared from the cells and used to detect the levels of phosphorylated and total ERK1/2 and DR4 by Western blot analysis. The indicated proteins were quantified, and each was previously normalized to the level of GAPDH. Data are means ± SD for 3 independent experiments. *P < 0.05, **P < 0.01* vs.* control.

Bestatin exhibits specific biological functions *via* the MAPK and PI3K signaling pathways in certain tumor cell lines^5,8^; therefore, we examined these pathways in our experimental system. According to the results from Western blot analysis, in comparison to that in control cells, ERK1/2 phosphorylation was markedly decreased in all tested tumor cells exposed to bestatin in a time- and dose-dependent manner (*P < 0.05; **P < 0.01) (**Figure 5A and 5B**). Moreover, the expression of phosphorylated ERK1/2 was prominently decreased by WM15 treatment (**P < 0.01) (**Figure 5C**). Cells transfected with CD13 siRNA exhibited much lower levels of p-ERK1/2 than did cells transfected with siRNA control (*P < 0.05; **P < 0.01) (**Figure 5D**). Next, because down-regulation of p-ERK1/2 was identified in the presence of CD13 inhibitors, we investigated whether this effect might be associated with TRAIL-mediated cell death. As shown in **Figure 6A**, TRAIL alone very weakly suppressed the growth of the tested cells, whereas PD98059, an ERK inhibitor, enhanced the decrease in cell proliferation caused by TRAIL; however, TPA, an ERK activator, reversed the decreased cell viability rates in the TRAIL plus PD98059 group (**P < 0.01). These findings suggest that the enhanced inhibitory effect of TRAIL on tumor cell viability is tightly correlated with p-ERK1/2 down-regulation mediated by CD13 inhibition. In addition, we detected the expression of other signaling pathway proteins including NF-κB, IκBα, c-Jun, p38, and their phosphorylated forms; however, each showed inconsistent changes in tested cell lines (**Supplementary Figure S6**), thus indicating that they are not likely to be involved in fundamental molecular events in the modulation of the synergistic anti-tumor effects of TRAIL and CD13 inhibition.

On the basis of our results, CD13 inhibition augmented TRAIL’ s effect in decreasing tumor cell survival through DR4 activation as well as p-ERK1/2 down-regulation; thus, we reasoned that a possible link might exist between these pathways. Consequently, we examined whether the DR4 expression might be modulated by p-ERK1/2 down-regulation in tumor cells. As presented in **Figure 6B**, after a 24 h exposure, PD98059 significantly diminished the levels of DR4 protein, and this response was accompanied by suppression of ERK1/2 phosphorylation in A549 and MCF-7 cells (*P < 0.05; **P < 0.01). Although ERK inhibitor treatment alone indeed decreased the DR4 expression, the results were opposite from the increase in DR4 levels with CD13 inhibition, as shown in **Figure 3**, thus demonstrating that CD13 inhibition augments TRAIL/DR4-induced apoptosis in a p-ERK1/2-independent manner.

### Therapeutic efficacy *in vivo*

**Figure 7:**
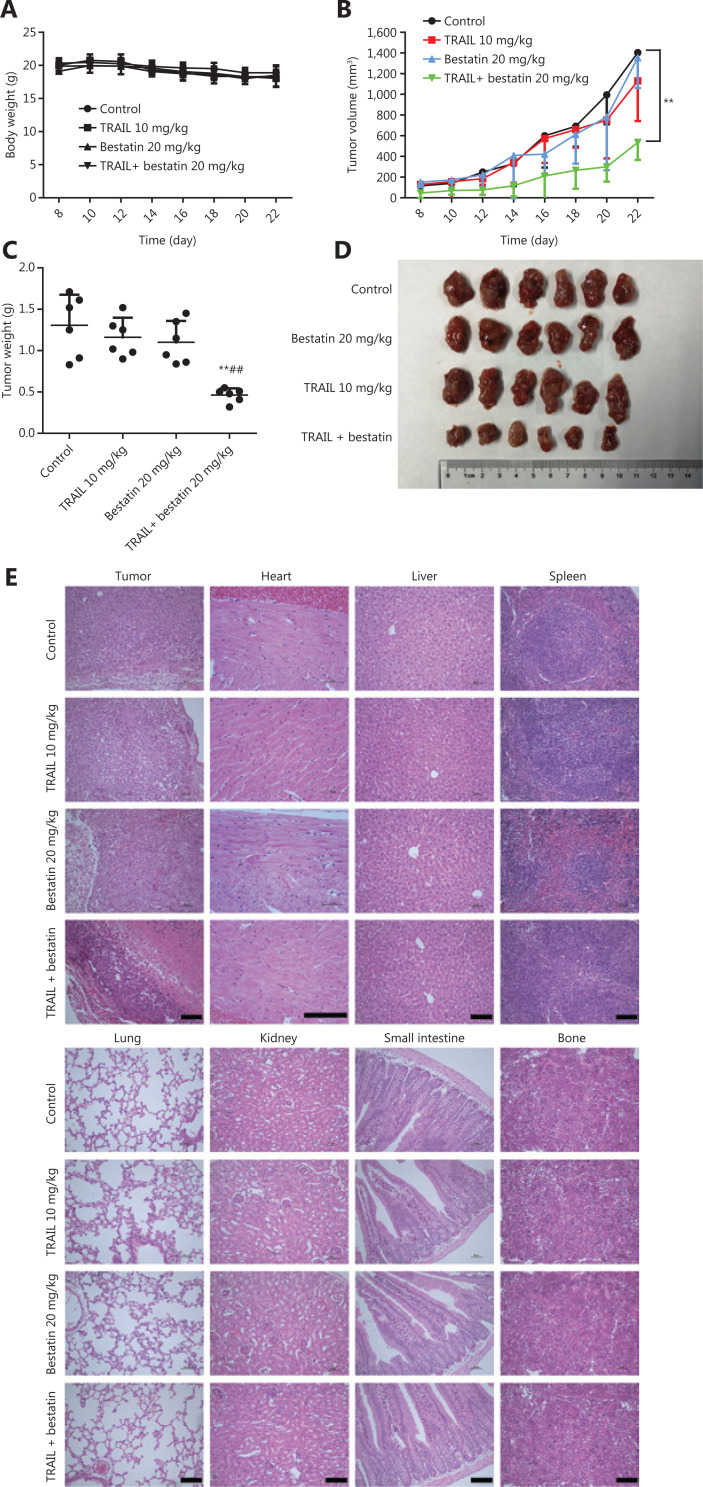
Bestatin and/or TRAIL inhibit the growth of xenograft tumors in nude mice. (A) Body weight changes in HT1080 xenograft-bearing nude mice. (*n* = 6). (B) Tumor growth curves of the HT1080 xenograft (*n* = 6). **P < 0.01 *vs*. control. (C) The tumor weights in the 4 groups. **P < 0.01 *vs.* control, ^##^*P* < 0.01 *vs*. TRAIL. (D) Representative photographs of the excised tumors from the 4 groups. (E) Histopathological examination of tumors and various organs of HT1080 xenograft-bearing nude mice. Histology was visualized under a microscope. The scale bar corresponds to 100 μm.

**Figure 8:**
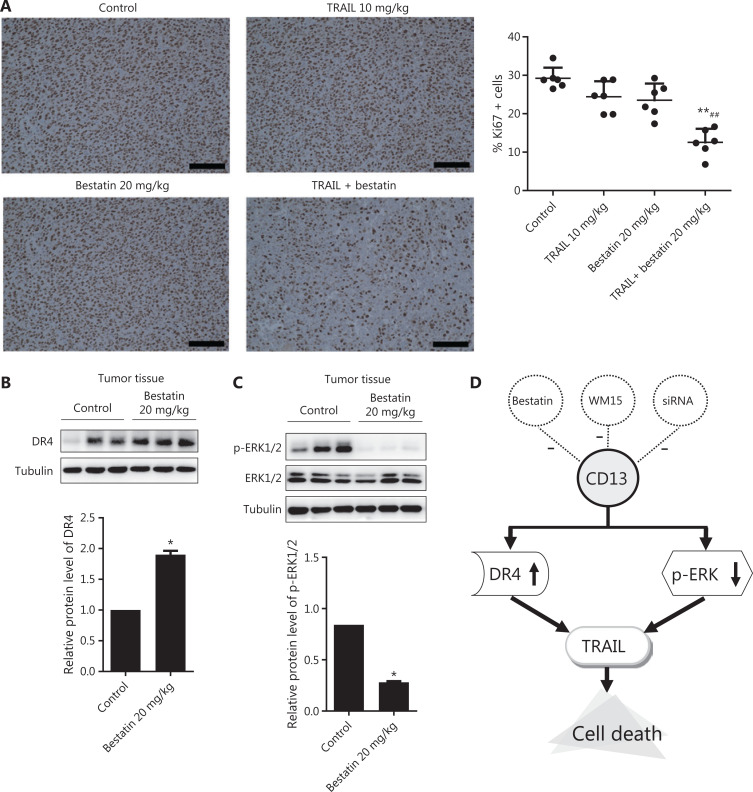
Immunohistochemical staining to determine the expression of Ki67, DR4, and p-ERK1/2 in tumor tissues of nude mice treated with bestatin and/or TRAIL. (A) Cells with brownish yellow particles were considered Ki67 positive in HT1080 xenografts. The percentage of cells with positive staining for Ki67 expression was normalized to that in the control group. *n* = 6. **P < 0.01 *vs*. control, ^##^*P* < 0.01 *vs.* TRAIL. The scale bar corresponds to 100 μm. The harvested tumors were lysed, and Western blot analysis was performed to detect the expression of DR4 (B), and phosphorylated and total ERK1/2 (C). The indicated proteins were quantified, and each was previously normalized to the level of tubulin. *n* = 3. *P < 0.05 *vs.* control. (D) Schematic presentation of the synergistic inhibitory mechanism.

Because of the synergistic anti-neoplastic effects of TRAIL and CD13 inhibition *in vitro*, we sought to determine the efficacy *in vivo* by using an HT1080 xenograft model, which has favorable tumorigenicity characteristics. All treatments were well tolerated by nude mice and caused no death, significant weight loss (**Figure 7A**), or toxicological changes in organs and tissues (**Figure 7E**). According to the data in **Figure 7B and 7C**, TRAIL plus bestatin treatment clearly enhanced the decrease in tumor volume and tumor weight, as compared with the control or single agent therapy results (**P < 0.01; ^##^*P* < 0.01). TRAIL or bestatin alone slightly suppressed tumor growth by 10.98% and 15.71%, respectively, whereas a 64.62% decrease in tumor growth was surprsingly observed in the TRAIL plus bestatin treatment group, and the CDI was 0.47, thus indicating a strongly synergistic drug-drug interaction. Furthermore, these effects were apparent in the morphology and size of tumor xenografts (**Figure 7D**). Immunohistochemical analysis of Ki67 also revealed markedly decreased proliferation (**P < 0.01) (**Figure 8A**). The expression of DR4 and p-ERK1/2 in the isolated tumor tissues was in agreement with the above findings *in vitro*: bestatin substantially increased the DR4 levels and decreased phosphorylation of ERK1/2 (*P < 0.05) (**Figure 8B and 8C**).

## Discussion

Cytotoxic process triggered by DR activation through binding to TRAIL are considered promising targets for anti-tumor therapy, owing to selective killing effects on tumor cells. Given the potential role of TRAIL, understanding the underlying mechanism, particularly by identifying how to render tumor cells susceptible to TRAIL induced cell death, has been a major focus of investigation. In this study, we discovered that inhibition of CD13 enhanced the decrease in cell proliferation and the increase in apoptosis induced by TRAIL, and both treatments synergistically suppressed the invasion and migration of tumor cells. To our knowledge, this is the first study demonstrating that CD13 inhibition cooperates with TRAIL in causing enhanced anti-tumor effects.

Five receptors are known to bind TRAIL: the membrane-bound receptors DR4, DR5, DcR1, and DcR2, and osteoprotegrin (OPG), a soluble receptor. TRAIL binding to only DR4 or DR5 leads to receptor trimerization and recruitment of Fas-associated death domain-containing protein, which cleaves PARP^22^. In our study, we observed that the killing effect of TRAIL in tumor cells was potentiated by CD13 inhibitors, owing to the increase in expression and cell surface of DR4 protein. MG132 further decreased the degradation of DR4 protein, thus preliminarily illustrating the participation of the ubiquitin-proteasome pathway in increasing DR4 protein stability in the presence of CD13 inhibition. That is, CD13 inhibition decreases proteasome activity and increases DR4 stability. In addition, because bestatin did not affect DR4 mRNA, we concluded that CD13 inhibition enhances DR4 expression at the protein level rather than the transcriptional level. Knockdown of DR4 attenuated cell death, and the increased PARP cleavage mediated by TRAIL had a cooperative effect with CD13 inhibition, thereby demonstrating that DR4 is critical in this cytotoxic process against tumor cells. In agreement with this finding, several agents including celecoxib^23^, retinoic acid^24^, and azithromycin^25^ have been documented to enhance the apoptosis induced by TRAIL through increasing the levels of DR4 or/and DR5. Interestingly, we did not observe a similar phenomenon for DR5; indeed, CD13 inhibitor treatment did not increase the cell surface levels of DR5 in the tested cells under the same conditions, thus suggesting that DR5 may not be functionally necessary for the synergistic anti-tumor effects of TRAIL and CD13 inhibition in this study.

DR4 has been shown to be up-regulated through a MEK-dependent pathway^26^. In our study, on the basis of the 2 main mechanisms of DR4-activition and p-ERK1/2 down-regulation involved in the cooperative effects of TRAIL/DR4 and CD13 inhibition, we determined the possible relationship between the 2 pathways described above. Indeed, PD98059, an ERK inhibitor, significantly diminished the expression of DR4 protein. Although the study was similar to research by Drosopoulos^26^, the results of that study are opposite from the elevation of DR4 level observed in the current study. The discrepancy is likely to be due to the role of CD13 inhibition in mediating increased DR4 protein expression and cell surface levels, and suppressed protein degradation, thus overwhelming the influence of p-ERK1/2 through additional regulatory mechanisms and eventually augmenting TRAIL/DR4-induced cell death in tumor cells.

Previous studies have highlighted that resistance to TRAIL monotherapy in clinical trials is the major obstacle and limitation of TRAIL-based treatment, in agreement with findings regarding DR5 cellular localization^27^, anti-apoptotic proteins of the Bcl-2 family^28^, the EGFR signaling pathway^29^, the multidrug transporter P-glycoprotein (Pgp)^30^, and Smad (Sma-Mad) 7 protein^31^. In addition, TRAIL sensitivity is inversely associated with cellular FLICE-like inhibitory protein (cFLIP) in breast cancer stem cells, as evidenced by the cytotoxicity alleviation in the presence of cFLIP overexpression^32^. The ERK1/2 pathway mediates cFLIP expression, thus affecting TRAIL sensitivity^33^. We discovered that p-ERK1/2 levels were altered during CD13 inhibition, thus facilitating tumor cell sensitization to TRAIL-induced killing, in agreement with reports that CD13 inhibitor decreases the phosphorylation of MAPK^5,8^. Thus, we speculated that the interaction of TRAIL/DR4 with CD13 inhibition might potentially affect the modulation of cFLIP *via* the ERK1/2 pathway, together with changes in other important factors, thus overcoming the resistance to TRAIL. We will investigate this possibility in future research.

Because the CD13 inhibitor bestatin is an approved drug for adjuvant treatment of cancer, our finding that inhibition of CD13 activity with small molecules and monoclonal antibodies significantly facilitated tumor cell susceptibility to TRAIL-mediated killing, both *in vitro* and *in vivo*, may feasibly provide an effective way to enhance TRAIL- or DR4 activation-induced death of tumor cells, even in clinical settings.

## Conclusions

The current study provides the first reported evidence that CD13 inhibition cooperates with TRAIL in enhancing DR4 activation-mediated cell death *via* the up-regulation and stabilization of DR4 in a p-ERK1/2-independent manner (**Figure 8D**). Our findings highlight the potential utility of CD13 inhibition for TRAIL/DR4-based therapy against neoplastic diseases.
