# Supplementary material for: CD13 inhibition augments DR4-induced tumor cell death in a p-ERK1/2-independent manner
Source: Cancer Biol Med. 2021 Jun 15;18(2):569–86. doi: 10.20892/j.issn.2095-3941.2020.0196 (PMC8185856; doi:10.20892/j.issn.2095-3941.2020.0196)
Supplement: Supplementary file 1 [file cbm-18-569-s001.pdf]

Supplementary materials

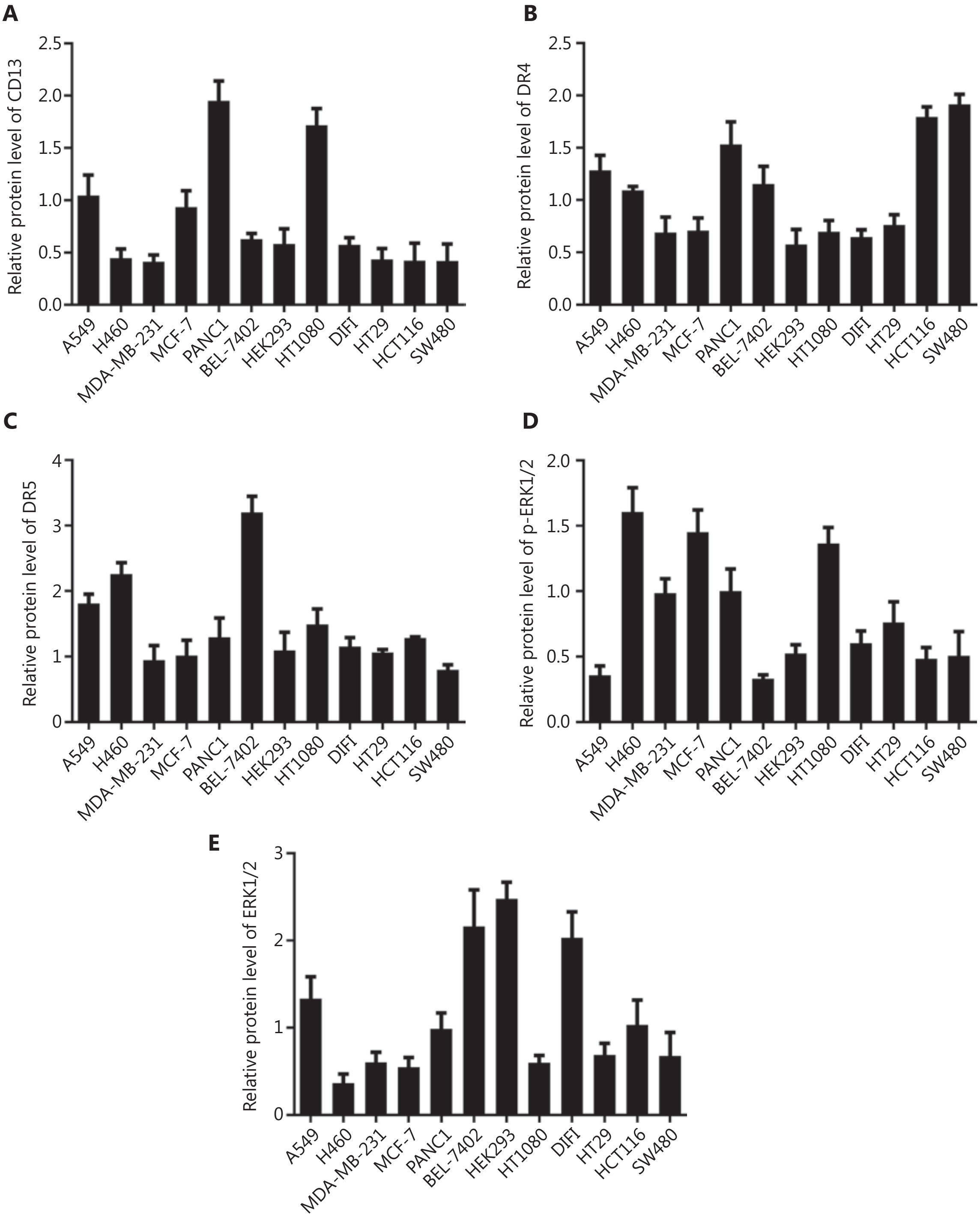

Figure S1 Expression levels of CD13, DR4, DR5, p-ERK1/2, and ERK1/2 in the indicated tumor cell lines. After Western blot analysis, CD13 (A), DR4 (B), DR5 (C), p-ERK1/2 (D), and ERK1/2 (E) were quantified, and each was normalized to the level of GAPDH. Data are means ± SD for 3 independent experiments.

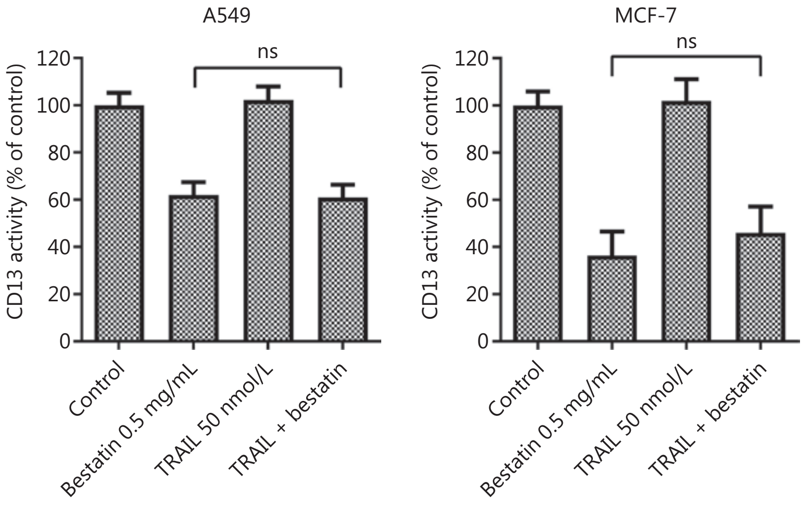

Figure S2 The effects of bestatin or/and TRAIL on CD13 enzyme activity. The indicated cell lines in 96-well plates were pretreated with the indicated agents (20 µL) for 1 h, and this was followed by treatment with L-alanine 4-nitroanilide hydrochloride (20 mmol/L) as a protease substrate for 1 h. The CD13 activity rates were normalized to those of the control group (untreated cells). Data are means ± SD for 3 independent experiments.

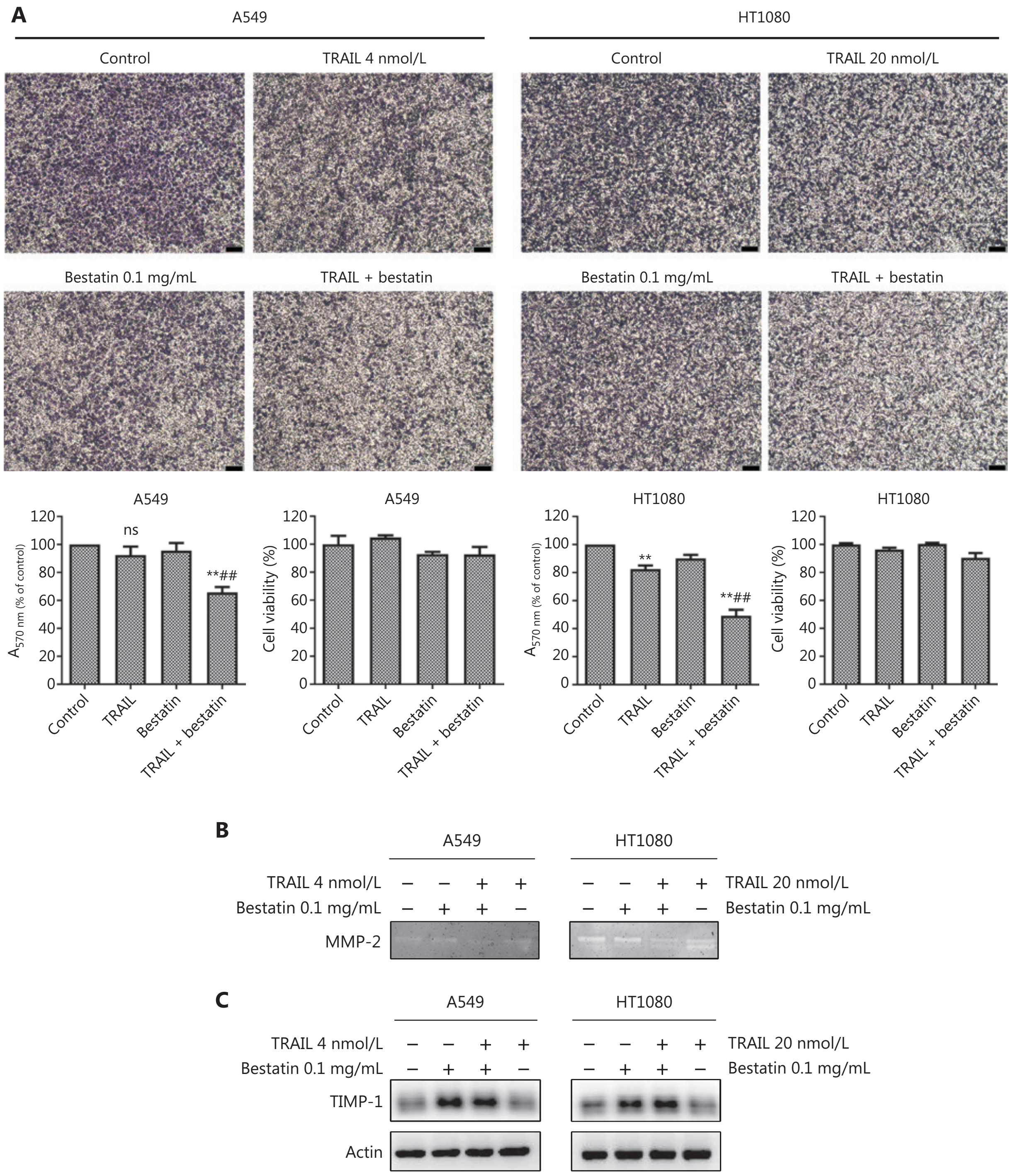

Figure S3 CD13 inhibition cooperates with TRAIL in enhancing the anti-invasion effect on tumor cells. (A) Cell invasion responses to bestatin (0.1 mg/mL) and/or TRAIL (A549: 4 nmol/L, HT080: 20 nmol/L) were determined with Transwell assays. The absorbance value of each sample was measured at 570 nm and normalized to the control group values (untreated cells). Data are means ± SD for 3 independent experiments. Ns and $^{**}P < 0.01$ *vs*. control, $^{\#\#}P < 0.01$ *vs*. TRAIL. The scale bar corresponds to 100 μm. After treatment at the indicated concentrations, the enzymatic activity of MMP-2 was determined with gelatin zymography assays (B) after 24 h and the expression of TIMP-1 was detected by Western blot analysis (C) after 12 h.

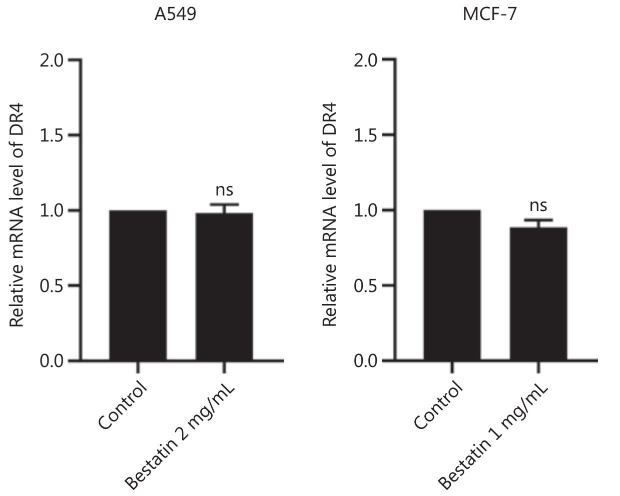

Figure S4 The effect of CD13 inhibition on DR4 mRNA expression. The indicated cell lines were treated with the indicated concentrations of bestatin for 4 h. The mRNA levels were normalized to GAPDH mRNA expression. The relative mRNA levels are shown as fold changes relative to the control group values (untreated cells). Data are means ± SD for 3 independent experiments.

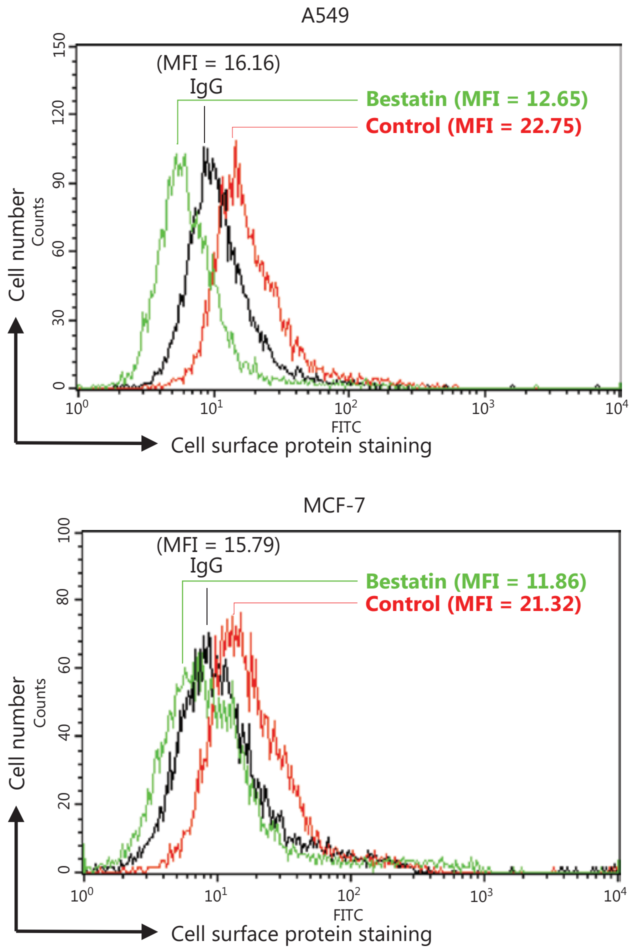

Figure S5 CD13 inhibition decreases cell surface DR5. The indicated cell lines were treated with bestatin (A549: 4 mg/mL, MCF-7: 2 mg/mL) for 24 h and then harvested for staining of DRs and subsequent flow cytometric analysis of cell surface DR5. The control cells were stained with a matched control FITC-conjugated IgG isotype antibody or FITC-conjugated anti-DR5 antibody, and the bestatin-treated cells were stained with FITC-conjugated anti-DR5 antibody. MFI for each sample is indicated.

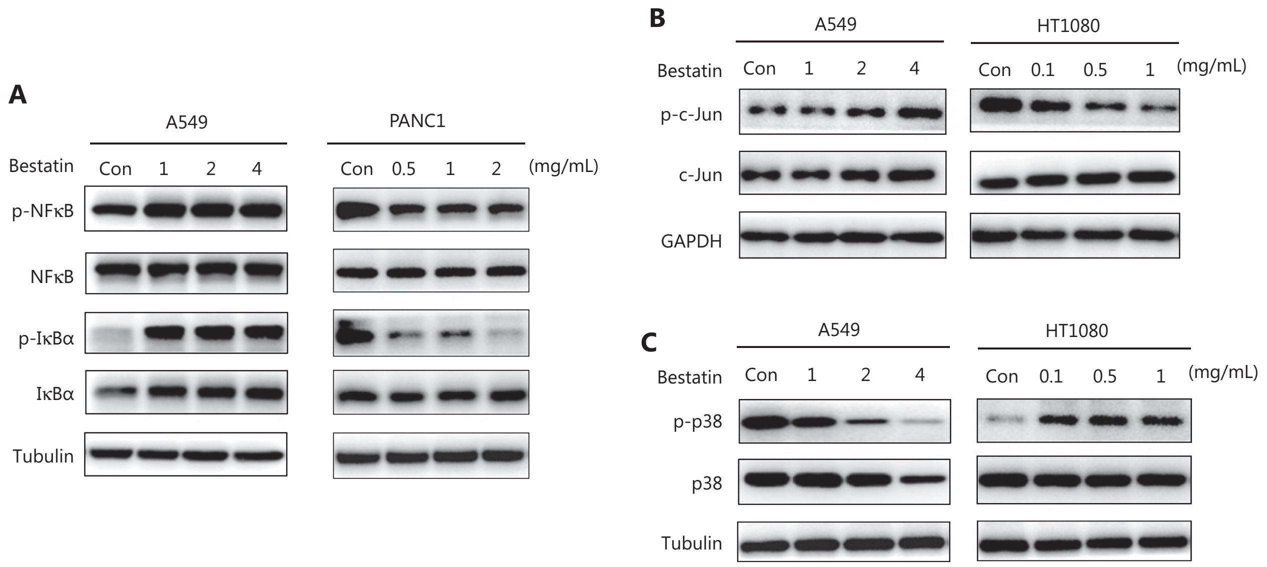

Figure S6 The effect of CD13 inhibition on the expression levels of NF-κB, IκBα, c-Jun, p38, and their phosphorylated forms. The indicated cell lines were treated with the indicated concentrations of bestatin for 24 h. After the indicated treatments, whole cell lysates were prepared from these cells and used to detect the levels of phosphorylated and total NF-κB (A), phosphorylated and total IκBα (A), phosphorylated and total c-Jun (B), and phosphorylated and total p38 (C) through Western blot analysis.
